# Effect of Adding MoDTC on the Properties of Carbon Black Rubber and the Friction and Wear of Metal during Mixing Process

**DOI:** 10.3390/ma13051071

**Published:** 2020-02-28

**Authors:** Yiren Pan, Deshang Han, Lin Zhu, Meng Zhang, Huiguang Bian, Chuansheng Wang, Wenwen Han

**Affiliations:** 1 College of Electromechanical Engineering, Qingdao University of Science and Technology, Qingdao 266061, China; pyr90hot@163.com (Y.P.); 17853253362@163.com (D.H.); qustzhulin@163.com (L.Z.); zhangmeng96710@163.com (M.Z.); bianhuiguang@163.com (H.B.); 2 Shandong Provincial Key Laboratory of Polymer Material Advanced Manufactorings Technology, Qingdao University of Science and Technology, Qingdao 266061, China; 3 Academic Division of Engineering, Qingdao University of Science & Technology, Qingdao 266061, China; 4 National Engineering Laboratory for Advanced Tire Equipment and Key Materials, Qingdao University of Science and Technology, Qingdao 266061, China

**Keywords:** mixing process, carbon black rubber compound, MoDTC, metal, friction and wear

## Abstract

The gap between the rotor and the mixer chamber wall is an important factor in determining filler dispersion in rubber compounds. The inner wall of a mixer will wear after working for a long time, which will cause poor filler dispersion and affect the quality of rubber products. In this study, MoDTC was added to carbon black as a kind of filler, and the effect on filler dispersion, the properties of the rubber product, and the friction and wear of rubber and metal in the mixing process were examined. Experimental data showed that after adding 3 phr of MoDTC, carbon black dispersion was greatly improved, the curing time was shortened, and the performance remained stable. It was also found that the Mo element of the compound with 3 phr MoDTC dispersed better than that of the other compounds. Most importantly, adding 3 phr of MoDTC greatly reduced the amount of wear on the metal during the mixing process. However, the opposite effect occurred when the MoDTC content was high. The method proposed in this study can not only improve filler dispersion in rubber but also reduce metal wear to prolong the service life of the mixing chamber when applied to an actual mixing process.

## 1. Introduction

**Figure 1:**
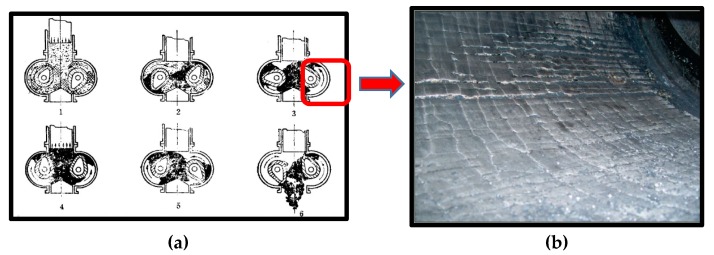
Mixing process and chamber wear diagram: (**a**) chamber structure and (**b**) photos of chamber wear.

The gap between the mixer wall and the rotor is an important parameter of mixers, which affects the rubber quality and compound dispersion. Studies have shown that the factory-set gap between the rotor and mixer wall is the most optimum value [1,2,3,4]. In particular, carbon black (CB) has widely been used as a principal reinforcement element in the rubber industry for more than a century [5], and a large amount of carbon black is added to the tire formula to improve tire performance. However, other fillers are also added or increased in the formula in order to enhance certain characteristics of rubber products according to the product performance requirements [6,7,8], which will cause friction and wear with the mixer wall [9], as shown in Figure 1 [10]. When the wear reaches a certain extent, the increased clearance between the mixer wall and the rotor will cause uneven dispersion of fillers, which will ultimately affect the comprehensive mechanical properties of the rubber.

**Figure 2:**
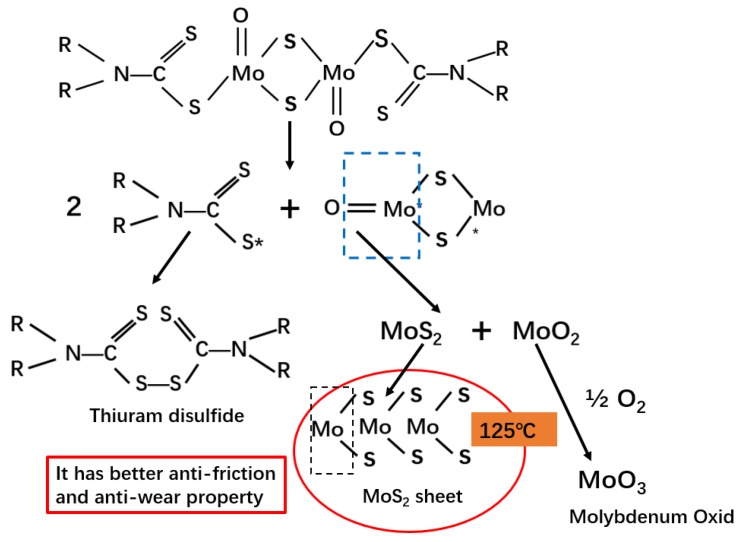
The generation of molybdenum disulfide (MoS_2_) from molybdenum dithiocarbamate (MoDTC).

Recent studies have reported that the main repair method is machine halt and do surfacing welding. However, surfacing weld can crack, and surface oxidation may occur due to the different technology and welding materials. In addition, if there is poor combination between surfacing welding and raw materials, more serious wear and tear will be generated, which may even flake into the mixed rubber and affect the performance and quality of the rubber product. Hence we studied adding appropriate amount of anti-friction agent to the formula to reduce the wear between rubber and mixer chamber wall during the mixing process. Many studies have shown that molybdenum dithiocarbamate (MoDTC) has the best antifriction effect at 250 °C while losing its antiwear effect at 320 °C. However, MoDTC decomposes into molybdenum disulfide (MoS_2_) at 125 °C, which has better antiwear performance [11,12]. In this study, we chose solid MoDTC not only because of its good performance but also because the solid particles are more easily dispersed in the mixing process. Figure 2 shows the generation of MoS_2_ from MoDTC. Other studies have reported that the addition of MoS_2_ can significantly improve CB dispersion in solution styrene butadiene rubber (SSBR) [13,14,15]. Therefore, a certain amount of MoDTC can be added to the tire formulation to promote carbon black dispersion and reduce the wear of the mixer chamber wall during mixing process.

In the present work, a small amount of antifriction agent was added to the existing formula as a new method for extending the service life of mixing chambers. Solid MoDTC was selected as the antifriction agent because it can decompose into MoS_2_ in the process of friction and wear over 125 °C, which can play a role in reducing wear. The optimal amount of anti-wear agent to be added without changing the comprehensive mechanical properties of rubber was established, and the influence of the antifriction agent on friction and wear in the mixer chamber was studied in order to prolong the service life of mixing chamber.

## 2. Materials and Methods

### 2.1. Materials

Natural Rubber (NR): TSR20, Thailand 20# Standard Rubber, Thailand; Polymerized Styrene Butadiene Rubber (SBR): RC2557S with 57% the content of vinyl, and ML (1 + 4) @100 °C is 54, products of PetroChina Dushanzi Petrochemical Company, Dushanzi, China; Cis-Polybutadiene (BR): BR9000, products of PetroChina Dushanzi Petrochemical Company, Dushanzi, China; Carbon black (CB): N234, products of Cabot America; Silica: Silica115MP, products of Rodia; Silane Coupling Agent: Si69mix, products of Nanjing Shuguang Chemical Group, China; 1,3-Diphenylguanidine(DPG): Guiechem, China; Molybdenum Dithiocarbamate (MoDTC): Hangzhou Shitean chemical limited company, China; Sulphur (S): products of PetroChina, China; N-cyclohexylbenzothiazole-2-sulphenamide (CZ): products of Guiechem, China; The protective system, the activation system are all qualified products approved by the industry.

### 2.2. Experiment Formulation

The formula used in this study is shown in Table 1. Mixing was carried out using the Hackmie Machine developed by Qingdao University of Science and Technology. Fill factor and rotor speed was kept constant at 0.75 and 80 rpm, respectively. In this experiment, rubber and compound materials were mixed as per the values outlined in Table 2.

**Figure 3:**
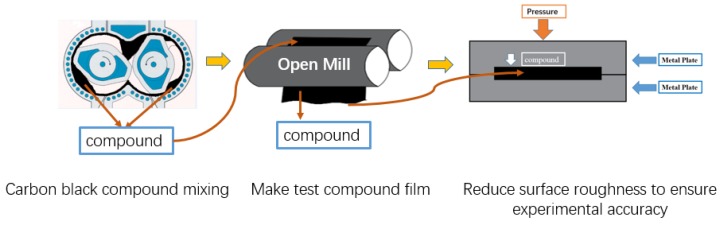
Sample preparation process.

Because of the requirements relating to the shape of the rubber samples and to ensure accuracy of the experiment, the test compound samples were made through pressurized steel plate. The friction coefficient is also related to the surface roughness. Therefore, before the friction test, we put the compound samples into a grinding tool to make the surface roughness as smooth as possible to reduce test error. The production process of the test piece is shown in Figure 3.

### 2.3. Experimental Process

A rubber processing analyzer (RPA 2000) was used to test the dynamic rheological properties of the four systems. Strain scanning of the mixing rubber was as follows: frequency 1 Hz, temperature 60 °C, strain range 0.1 °C. For rubber hardness, the determination of indentation hardness was done by means of a durometer (shore hardness). The dynamic strain was measured by dynamic mechanical analysis (DMA; GABOMETER-150, GABO Germany). The tensile mode was used in temperature scanning; the frequency was 10 Hz, and the heating rate was 2 °C/min. Tensile properties were tested using the UT-2060 tensile force testing machine produced by Taiwan U-CAN Technology (U-CAN, China). The metal sample used for the friction test was the same as the material used in the Hackmie mixing chamber.

**Figure 4:**
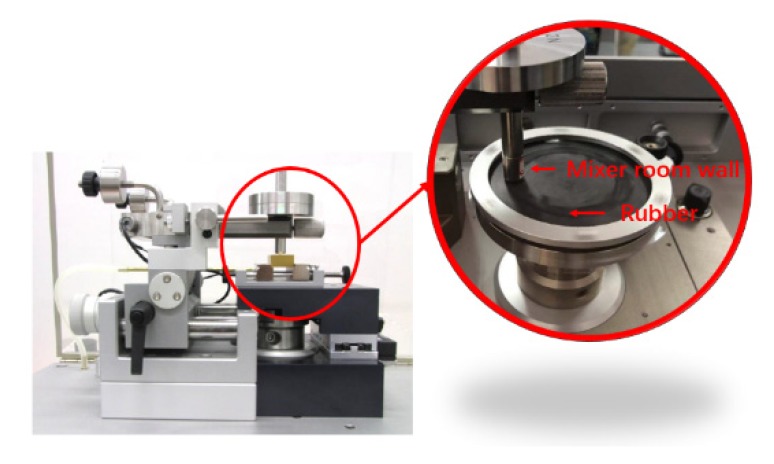
Equipment improvement image.

Friction and wear experiments between carbon black rubber composites and metal were carried out by Tribometer of Antor Paar, and TRB test for short (show in Figure 4). After the calibration, we chose the column plate model. Based on the calculation, the experimental pressure was set as 5 N, and the experiment time was set as half an hour. In order to accurately test the friction between the rubber and the wall of the mixing chamber during the mixing process, the mechanical testing part of the equipment was improved to make the material of the test contact surface the same as that of the mixing chamber. The friction test joints were replaced many times to repeat the test to ensure accuracy of the experiment.

Three-dimensional morphology test using Olympus 4500 was carried out to observe and measure the friction and wear of the metal surface. The wear amount was obtained by reducing the test volume.

## 3. Results and Discussion

### 3.1. Effect of Adding MoDTC on CB Compound Performance

**Figure 5:**
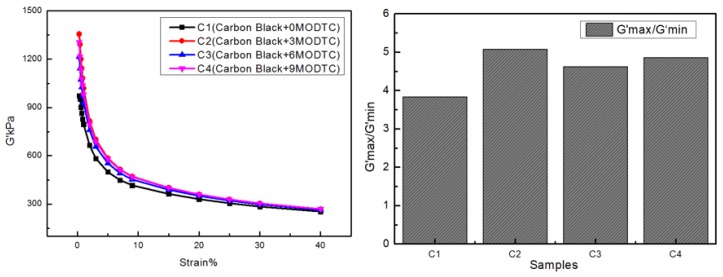
Effects of MoDTC on carbon black (CB) dispersion.

In carbon black rubber compounds, the dispersion state and the network of fillers play critical roles in determining the final properties of the composite. Figure 5 depicts the effects of MoDTC on CB dispersion and network in the composite. The Payne effect in RPA is usually used to reflect filler dispersion. Wang [16] elaborated this from the thermodynamics and dynamics viewpoints of filler network formation. The degree of filler network structure can be measured according to the elastic modulus (G′) strain curve. When the strain increases, the packing network is destroyed rapidly, and the G′ decreases rapidly; this is the Payne effect.

Figure 5 shows the effects of MoDTC on CB dispersion as measured by RPA 2000. We used G’ at high strain/G’ at low strain as an important parameter to research the Payne effect. This can also reflect the carbon black dispersion; the higher the G’ ratio, the better the carbon black dispersion. From the four sets of data, it was found that the G’ ratio obtained by adding 3 phr of MoDTC was the highest, and the G’ ratio calculated at C3 and C4 was higher than that of C1 without the addition of MoDTC. This indicates that MoDTC can promote the dispersion of carbon black. However, when the amount of MoDTC continued to be added, the G’ ratio showed a downward trend. This phenomenon can be explained by the fact that MoDTC in too high a proportion will lead to skid between rubber and metal, which affects the dispersion of filler in the rubber. More MoS_2_ will be produced from MoDTC with the increase of mixing temperature, and this is the reason MoDTC produces lubrication at high temperatures.

The cure characteristics of the compounds are exhibited in Table 3. ML can represent the fluidity of the rubber, MH can represent the shear modulus, hardness, elongation strength and cross-linking density of the rubber, tcx can represent the time required for the sample to reach a certain degree of vulcanization. It is evident that both MH and ML tended to increase with little change, while tc10 and tc50 had a declining trend with the increase of MoDTC. The optimum cure time (tc90) of the four compounds showed a downward trend. This phenomenon can be mainly attributed to the increase in the sulfur element with the increase of MoDTC, which promotes the generation of sulfur bond and shortens the curing time. Compared with tc90 of C1 and C2, the effect of adding MoDTC on curing time was more obvious.

Table 4 summarizes the mechanical properties of the vulcanizates. The hardness was not significantly affected by increased amount of MoDTC. 10% Tensile Modulus has a strong relationship with hardness. Compared with the 10% tensile modulus value of C1 and C2, the difference between them is small, which shows that adding 3 phr MoDTC has little effect on rubber hardness. The interaction between filler and rubber can be characterized by 100% tensile modulus. It can be seen that the 100% tensile modulus of C2, C3 and C4 shows an upward trend. However, compared with the other two groups, the upward trend of C2 was the most obvious. It can be concluded that adding 3 phrs of MoDTC can promote the carbon black dispersion in rubber. However, adding too much MoDTC will reduce the effect of carbon black dispersion in rubber. 300% tensile modulus can represent composite action both filler-rubber and rubber-rubber. Compared with the four groups, the rising trend of C2 was the most obvious, which showed that good carbon black dispersion and high curing crosslinking density. MoS_2_ was produced by MoDTC at 125 °C which cause vulcanization crosslinking density increased by the increasing sulfur elements, and 300% Tensile Modulus show upward trend. Comparing the four compounds, the tensile strength showed a downward trend, and C4 showed a more significant decline than the first three compounds. The rubber abrasion data showed the abrasion value of the four compounds saw little change. Combined with the data of the Payne effect, a very interesting phenomenon was obtained, that is, the MoDTC had an effect on the dispersion of packing, while it had little effect on rubber abrasion.

**Figure 6:**
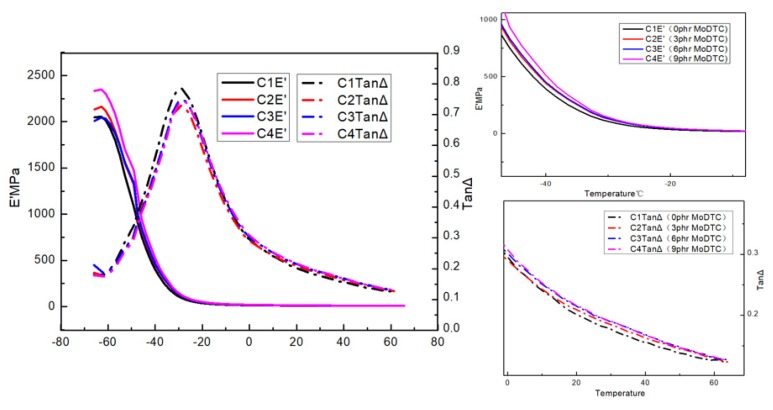
The dynamic mechanical analysis (DMA) E’ and tanδ curves of the four formulas.

Figure 6 shows the DMA E’ and tanδ curves of the four formulas. The DMA test can explore the molecular dynamics of rubber in response to wet sliding and rolling resistance. It is an important method to research the dynamic performance of rubber products or semi-finished products. The temperature corresponding to the maximum value of tanδ curve is usually used as the glass transition temperature of polymer. The values of tanδ at 0 °C and −20 to 20 °C can predict the wet sliding resistance, while tanδ at 40 and 60 °C can show the rolling resistance.

Figure 6 illustrates the effect on loss factor (tanδ) and E’. The glass transition temperature (Tg), tanδmax, and tanδarea extracted from Figure 6 are summarized in Table 5. The tanδ and E’ curves of the four formulas of the vulcanizates showed little change. The tanδ at 0, 40, and 60 °C were relatively stable with a small range of fluctuation. This is understandable because increasing amount of MoDTC promoted the dispersion of carbon black, enhanced the strength of the rubber–filler interaction, and led to an increase in the limitation of molecular movement. A comparison of experimental data also showed that, due to the increase of mixing temperature, MoS_2_ produced by MoDTC had no significant influence on the roll resistance and wet slip resistance of the rubber.

**Figure 7:**
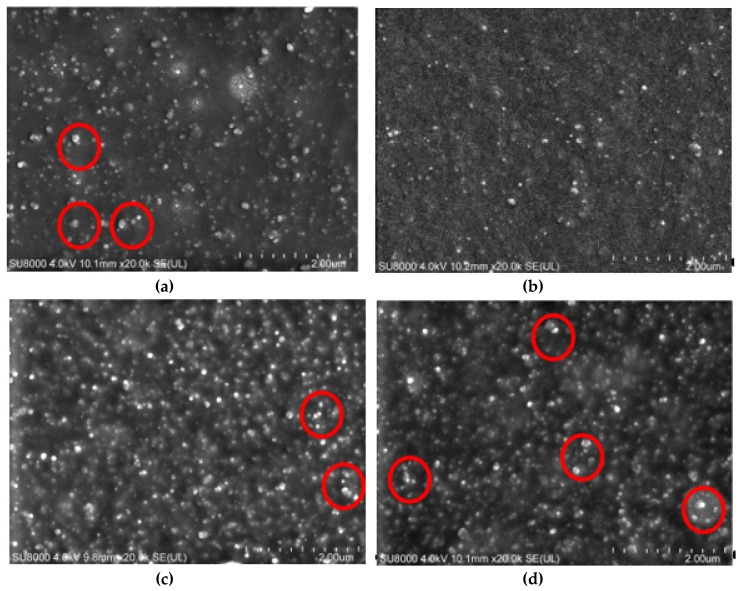
SEM images of the four compounds. (**a**) C1, (**b**) C2, (**c**) C3, (**d**) C4.

The SEM images of the four compounds are shown in Figure 7. From the SEM images, it can be seen that CB existed in rubber with different size aggregates. Comparing the SEM images of the four compounds, it could be seen that uneven and larger CB aggregate dispersion existed in C1 with no MoDTC. There were few carbon black aggregates in C2. In C3, numerous small carbon black aggregates appeared, while large carbon black aggregates were obvious in C4. Combined with the comprehensive analysis of the Payne effect measured by RPA, we believe that the addition of MoDTC can promote carbon black dispersion. However, when the amount of MoDTC is too much, MoS_2_ will be produced when the temperature reaches 125 °C during the mixing process, and the lubricating effect will make the rubber slip between the rotor and the refining chamber, which will weaken shear and tensile action. Therefore, carbon black aggregation will increase and therefore hinder carbon black dispersion.

**Figure 8:**
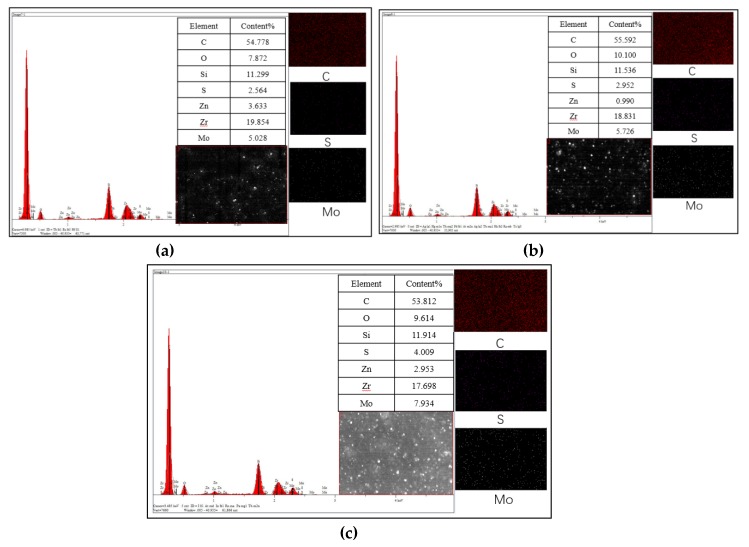
Elemental analysis of SEM. (**a**) C2, (**b**) C3, (**c**) C4.

Figure 8 is the elemental analysis of SEM, including test section images and analysis of element content. In addition to exploring the influence of MoDTC on carbon black dispersion, we also studied the effect of different amounts of MoDTC on its own dispersion during mixing.

Looking at the carbon element dispersion image, it is obvious that, compared to the addition of 6 or 9 phr MoDTC, the addition of 3 phr MoDTC resulted in more even dispersion in the test area. The addition of 9 phr MoDTC resulted in obvious carbon element accumulation and poor dispersion.

By comparing the dispersion of the Mo element in C2, C3, and C4 in the test area, it was found that the Mo element of the compounds with 3 and 6 phr MoDTC were evenly distributed. However, careful observation of the Mo element aggregation of C2, C3, and C4 showed that, as the amount of MoDTC increased, the Mo element had obvious accumulation. This was the case in C3 and especially in C4, where the Mo accumulation was greater. The experimental data show that the addition of too much MoDTC in the formula not only has an impact on carbon black dispersion during the mixing process but also has an impact on the dispersion of MoDTC itself.

### 3.2. Effect of Adding MoDTC on Friction and Wear between Rubber and Metal during Mixing

When the mixing temperature reaches 125 °C, MoDTC begins to decompose to MoS_2_. In previous studies, we have found that the wear is relatively severe in the last stage of mixing, while carbon black is generally discharged at 140–145 °C during the mixing process. Therefore, we took the rubber sample at 125–135 °C and set the TRB test temperature to 135 °C. This was aimed at exploring the effect of MoDTC on friction and wear between carbon black rubber and metal during mixing.

**Figure 9:**
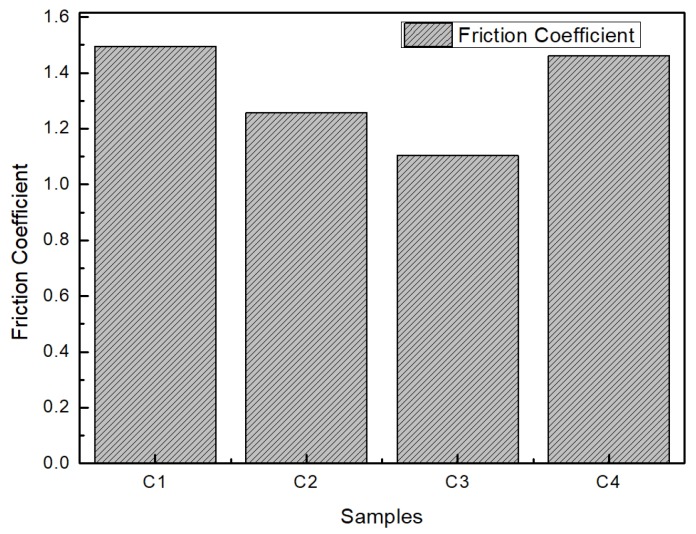
Average friction coefficient.

Figure 9 shows the friction coefficients between the four compounds and metal. The trend line shows that the friction coefficient gradually decreased with the increase in the amount of MoDTC added but suddenly increased with the addition of 9 phr MoDTC. The increased friction coefficient of C4 was almost the same as that of C1 without MoDTC and metal friction. Combined with the content and dispersion of the Mo element measured in Figure 8, it was found that excessive addition of antiwear agent would make the rotor slip with rubber during mixing, resulting in weakened mixing capacity and seriously affecting the dispersion of carbon black and other small materials. Therefore, a large amount of antifriction agent will not further reduce the friction coefficient between the compound and the metal as expected but will rather produce the opposite effect.

**Figure 10:**
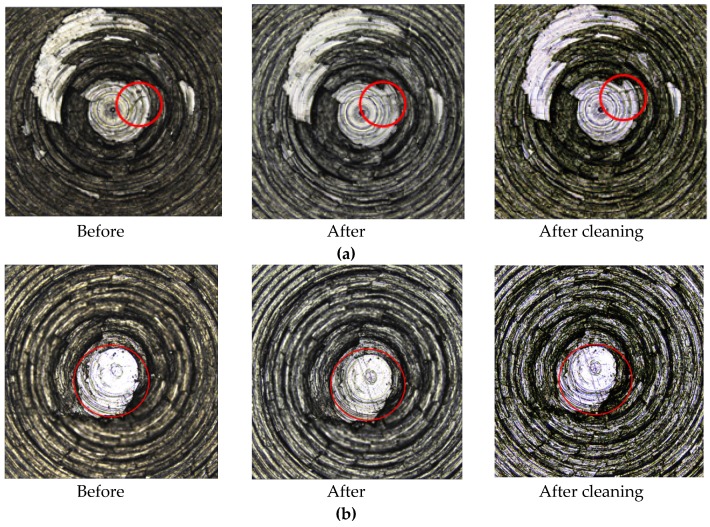

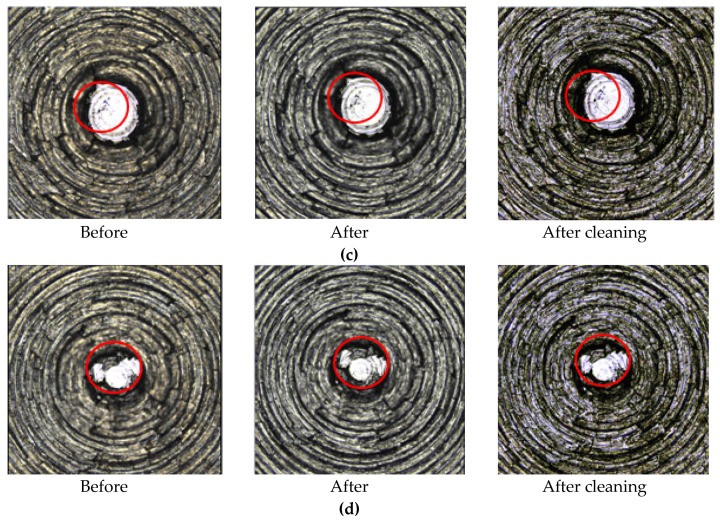
3D topography images of the metal. (**a**) C1, (**b**) C2, (**c**) C3, (**d**) C4.

Figure 10 were comparative surface morphology picture of the friction test between carbon black compound sample which were taken out at 125 °C–135 °C and the metal. The before images represented the metal surface morphology before friction with compound, the after one represented the metal surface morphology after friction with compound, and the after cleaning represented the metal surface morphology after ultrasonic cleaning with alcohol. The red mark is the comparison of surface morphology before and after friction.

**Figure 11:**
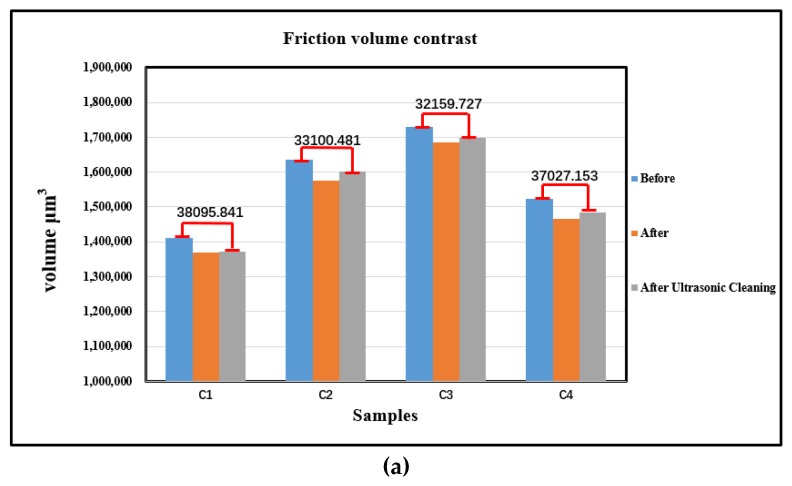

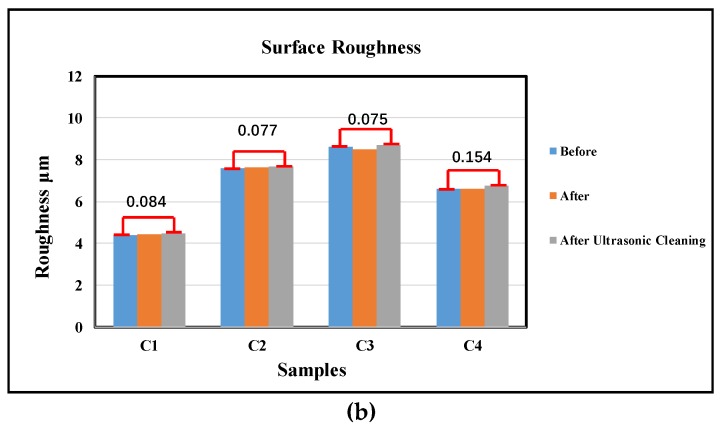
Friction volume contrast and surface roughness: (**a**) friction volume contrast, (**b**) surface roughness.

Figure 10 shows the metal surface topography measured by Olympus, and Figure 11 shows the volume and surface roughness of metal measured by Olympus. Based on the comprehensive analysis of Figure 10 and Figure 11, it was found that C1 without MoDTC had a tendency of crack growth on the metal surface after the friction test. Moreover, the volume loss and the surface roughness changed greatly before and after the friction test. Compared to C2 and C3 before and after friction, the metal surface morphology, the difference in volume, and the difference in roughness changed little. However, the volume and surface roughness of C4 with 9 phr MoDTC significantly increased before and after friction.

**Figure 12:**
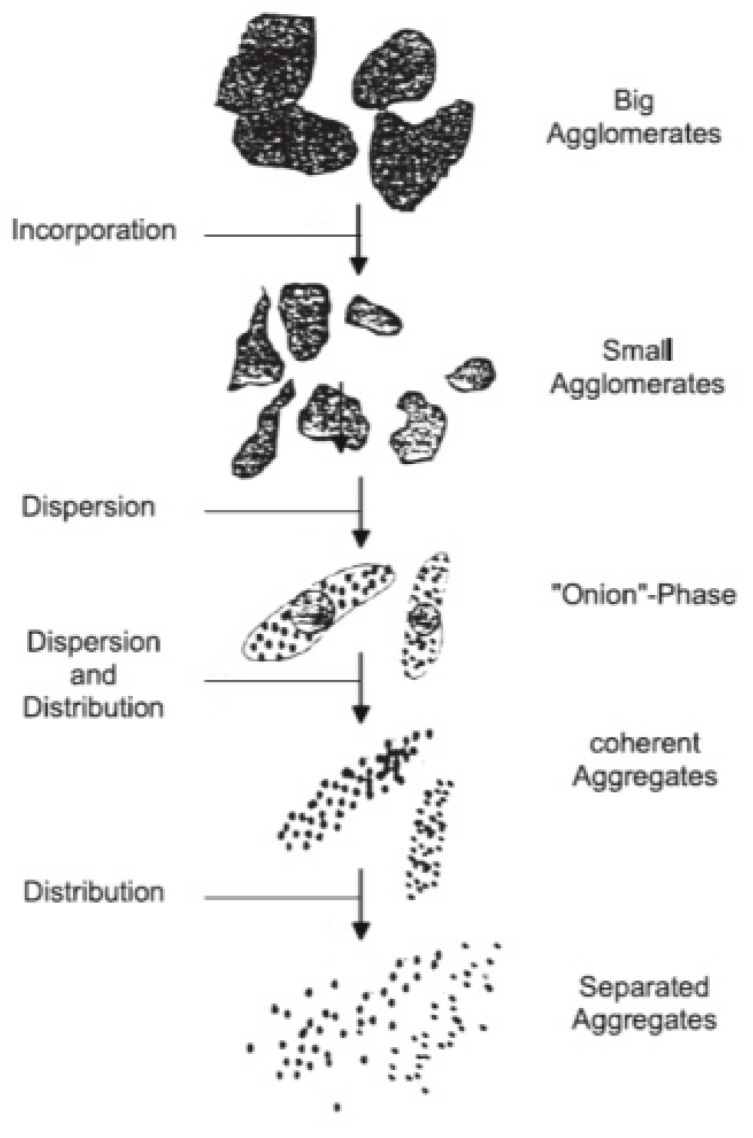
Carbon black dispersion process.

This can be explained by the fact that, when the mixing temperature reached 125 °C, carbon black with lubricating oil was absorbed by rubber. The reason for this phenomenon is that existing carbon black is mainly prepared by the furnace method, and some industrial oil is therefore retained on the surface, which means that there is a layer of oil film on the surface. At this time, carbon black is still in the stage of small agglomerates in the onion phase in the rubber, as shown in Figure 12. At this time, proper friction between the wall of the mixing chamber and the rotor can help carbon black dispersion and distribution in the rubber mixing process, and the appropriate lubrication effect can help the rubber flow in the mixing chamber, improving the speed of carbon black dispersion. However, when too much of MoDTC is added, it produces MoS_2_ with lubricating effect at 125 °C, and the tensile and shear effects of the rubber between the rotor and the wall of the mixing chamber will be weakened. The friction force provided by the rotor and the mixing chamber is weakened, which is conducive to mixing and dispersion. Carbon black still exists in the form of agglomeration, which will lead to intensified wear.

## 4. Conclusions

Certain amounts of MoDTC were added to the NR (Natural Rubber)/SBR(Polymerized Styrene Butadiene Rubber)/BR(cis-polybutadiene) formula with carbon black, which significantly improved the dispersion effect of carbon black in NR/BR/SSBR composites, increased the vulcanization rate, reduced the vulcanization time of rubber, and improved the comprehensive mechanical properties of rubber. According to the determination of elements, it was found that adding 3 phr MoDTC resulted in more easy dispersion in rubber. Considering the Payne effect, friction coefficient, three-dimensional morphology, and wear extent, a slight decrease in the friction coefficient and wear extent was found between the mixing rubber and the mixing chamber after the addition of appropriate amounts of MoDTC to the compound. However, the decrease in the friction coefficient did not affect the dispersion of carbon black and small materials in the mixing rubber, while the wear of the mixing chamber decreased. The opposite effect occurred when excessive MoDTC was added to the compound. The wear of the chamber wall was intensified, and the antiwear effect of MoDTC was reduced.

The experimental results show that adding 3 phr MoDTC to the formula not only improves filler dispersion during the mixing process but also reduces the amount of abrasion caused by the friction between the compound and the mixer chamber wall. This can be used as a new method to reduce the wear of mixer walls and prolong the service life of mixers.

## Figures and Tables

**Table 1:** Sample formula (unit: phr).

Raw Material	C1	C2	C3	C4
BR9000	25.5	25.5	25.5	25.5
RC2557S	81.81	81.81	81.81	81.81
TSR20	15	15	15	15
N234	60	60	60	60
Silica115MP	10	10	10	10
Si69mix	1.2	1.2	1.2	1.2
DPG	0.18	0.18	0.18	0.18
MODTC	0	3	6	9
S	1.3	1.3	1.3	1.3
CZ	1.8	1.8	1.8	1.8
Others	Protection system: 3.5 phr; activation system: 4 phr			

**Table 2:** Mixing conditions.

1.6 L Hake Mixer, 80 rpm, 75% FF ( Fill Factor)		
Time	T (°C)	Ingredients
Master batch		
0: 00	70	Polymers
0: 40	-	Chemical, carbon black
1: 10	-	Carbon black
2: 30	120	Sweep
4: 00	135	Sweep, sampling
5: 00	145	Discharge
Final mix (divided into two parts)		
Part 1: Put into a pressure plate container for friction and wear test		
Part 2: Curing system was added for performance testing		

**Table 3:** Cure characteristics of the compounds.

Test List	C1	C2	C3	C4
MH (N m)	18.78	19.64	19.60	19.65
ML (N m)	2.5	2.93	2.97	3.05
MH–ML (N m)	16.28	16.71	16.23	16.6
tc10 (min)	3.75	3.37	3.61	3.45
tc50 (min)	7.58	4.62	5.02	4.69
tc90 (min)	14.47	8.97	8.83	8.26

**Table 4:** Mechanical properties of the vulcanizates.

Test List	C1	C2	C3	C4
Hardness (ShoreA)	60.5	63.5	61	62.5
10% Tensile Modulus (MPa)	0.58	0.57	0.49	0.51
50% Tensile Modulus (MPa)	1.19	1.37	1.23	1.30
100% Tensile Modulus (MPa)	1.76	2.12	1.86	1.96
300% Tensile Modulus (MPa)	6.65	8.42	7.42	7.76
Tensile Strength (MPa)	15.71	15.14	15.27	14.75
Elongation at Break (%)	574.05	467.78	510.06	474.18
Rubber abrasion (%)	6%	7%	8%	8%

**Table 5:** Glass transition temperature (Tg), tanδmax, and tanδarea of the vulcanizates.

DMA	C1	C2	C3	C4
Tg	−28.68	−28.7164	−28.687	−28.7105
0 °C tanδ	0.2954	0.2900	0.30008	0.3078
−20 to 20 °C tanδ	14.2586	13.87645	14.4058	14.64935
40 °C tanδ	0.1555	0.1631	0.16797	0.1675
60 °C tanδ	0.1266	0.1300	0.12967	0.1314
